# Effect of Socioeconomic Status on Financial Toxicity: The Chain Mediating Roles of Social Support and Self‐Efficacy

**DOI:** 10.1002/cam4.71083

**Published:** 2025-07-25

**Authors:** Yan Liu, Pengfei Li, Boyu Liu, Yuantao Qi, Weimin Guan, Nan Zhang, Youhua Lu

**Affiliations:** ^1^ Shandong Cancer Hospital and Institute, Shandong First Medical University and Shandong Academy of Medical Sciences Jinan Shandong Province China; ^2^ School of Public Health Shandong Second Medical University Weifang Shandong Province China

**Keywords:** chain mediation effect, financial toxicity, lung cancer, self‐efficacy, social support, socioeconomic status

## Abstract

**Objective:**

Cancer patients experience severe financial toxicity, with the mechanisms influencing the relationship between socioeconomic status and financial toxicity in lung cancer patients remaining poorly defined. This study aims to investigate how social support and self‐efficacy mediate the association between socioeconomic status and financial toxicity among lung cancer patients in China.

**Methods:**

A survey of 755 lung cancer patients was conducted at a tertiary oncology hospital in Shandong Province, China, from October to December 2023, utilizing random sampling. Data collection included demographic and socioeconomic details, along with assessments of social support, self‐efficacy, and financial toxicity. Regression and Bootstrap analyses were used to explore the sequential mediating effects of socioeconomic status, self‐efficacy, social support, and financial toxicity.

**Results:**

(1) Significant correlations emerged among socioeconomic status, social support, self‐efficacy, and financial toxicity (*p* < 0.05). (2) Socioeconomic status was significantly associated with financial toxicity (*p* < 0.05). (3) Self‐efficacy mediated the relationship between socioeconomic status and financial toxicity (*β* = 0.203, *p* < 0.05), whereas social support did not exhibit a mediating effect in this relationship (*β* = 0.039, *p* = 0.194). (4) Social support and self‐efficacy had a chain‐mediated role in the relationship between socioeconomic status and financial toxicity in patients with multimorbidity (*β* = 0.072, *p* < 0.05).

**Conclusion:**

This study identifies social support and self‐efficacy as chained mediators that link socioeconomic status with financial toxicity among lung cancer patients. It is recommended that targeted interventions be implemented to increase social support for patients with lower socioeconomic status to mitigate financial toxicity.

## 1 Introduction

Cancer presents a significant public health challenge worldwide, particularly in China where, in 2022 alone, an estimated 4.82 million new cases and 2.57 million deaths occurred, with lung cancer being the most prevalent, accounting for approximately 1.06 million of the cases and 0.733 million of the deaths [1]. The impact of cancer extends beyond reduced survival rates and compromised quality of life; it additionally places substantial economic burdens on patients and their families [2]. The high costs associated with treatment, especially for long‐term and advanced care, frequently push families into financial hardship [3]. Furthermore, cancer patients often experience unemployment or reduced work capacity as a result of their illness, which exacerbates their financial stress [4].

With the advancement of oncology treatments, innovations such as new anticancer drugs and genetic testing have significantly improved patient outcomes and extended survival. However, these developments have also led to a substantial increase in the cost of cancer care [5]. Moreover, reduced productivity during recovery and the escalating costs of long‐term care further exacerbate the financial strain on patients and their families [6]. In 2012, Zafar et al. first introduced the concept of Cancer‐Related Financial Toxicity, comprehensively detailing the objective economic expenditures associated with cancer treatment, along with their consequent psychosocial distress, behavioral changes, and diminished quality of life [7, 8]. Financial toxicity (FT) refers to the adverse economic effects experienced by cancer patients due to escalating direct and indirect medical costs and diminished income during treatment or recovery [9]. This phenomenon is marked by both objective economic strain and subjective financial distress. Furthermore, financial toxicity is intricately linked to a deterioration in quality of life, aggravated symptoms, compromised medical adherence, and decreased survival rates, which may precipitate a cascade of negative repercussions [10].

Socioeconomic status (SES) is a multidimensional construct that is assessed by education level, occupational status, and income. These dimensions collectively reflect an individual's relative position in society and their capacity to access resources [11]. Higher socioeconomic status is generally associated with better health outcomes, as individuals in these brackets are more likely to access high‐quality healthcare, make healthier lifestyle choices, and benefit from broader social networks and superior living conditions [12]. Conversely, individuals with lower socioeconomic status experience greater economic pressures due to insufficient financial resources for managing costly treatments and related expenditures. This leads to higher levels of financial toxicity, which not only influences treatment decisions and outcomes but also exacerbates the psychological and emotional burdens for patients [13].

Social support, a critical psychosocial resource encompassing emotional solace, information exchange, and practical assistance from family, friends, and community, alleviates stress, enhances the capacity to cope with illness, and bolsters healthy behaviors [14]. Research indicates that adequate social support is closely linked to improved health outcomes, as it enables individuals to manage health challenges more effectively [15]. Furthermore, studies have demonstrated that a robust social support network can reduce financial toxicity in cancer patients by providing emotional support and potential resource sharing, which helps alleviate the economic pressures associated with treatment [16].

Self‐efficacy refers to an individual's belief in their capacity to manage life's various challenges, including health‐related issues [17]. Individuals with high self‐efficacy are more likely to engage in proactive health management behaviors, adhere more closely to medical advice, and effectively address the challenges posed by illness [17]. Research suggests that individuals with high self‐efficacy proactively manage their treatment during health crises, experience lower levels of financial toxicity, efficiently utilize available resources, and adapt to treatment demands. Furthermore, their intrinsic belief system indirectly mitigates the effects of economic stress by influencing their behavioral choices and coping strategies [18].

While extensive research has highlighted the impact of socioeconomic status on the financial toxicity experienced by cancer patients, the specific roles of social support and self‐efficacy in this process remain insufficiently understood, particularly within the sociocultural context of China [19]. There is a critical need to explore how social support and self‐efficacy can mitigate financial toxicity and to understand the pathways of their interaction with socioeconomic status. Moreover, although existing studies suggest that social support and self‐efficacy may mediate the effects of financial toxicity, these investigations have predominantly focused on simplistic mediation models [20]. There is a notable gap in the literature regarding how these variables might operate through a chain mediation effect, making the exploration of this complex interaction mechanism particularly crucial.

Although financial toxicity is widely recognized for its significant impact on the quality of life and treatment outcomes of cancer patients, research into effective social and psychological interventions to alleviate this burden is still lacking [19]. Specifically, there is an urgent need for further research on early intervention strategies designed to prevent the onset of financial toxicity. To address this research gap, this study aims to explore how social support and self‐efficacy jointly mediate the relationship between socioeconomic status and financial toxicity using a chain mediation model. By establishing a more comprehensive theoretical framework and empirical foundation, this study seeks to contribute to the development of effective intervention strategies.

## 2 Materials and Methods

### 2.1 Participants

**FIGURE 1:**
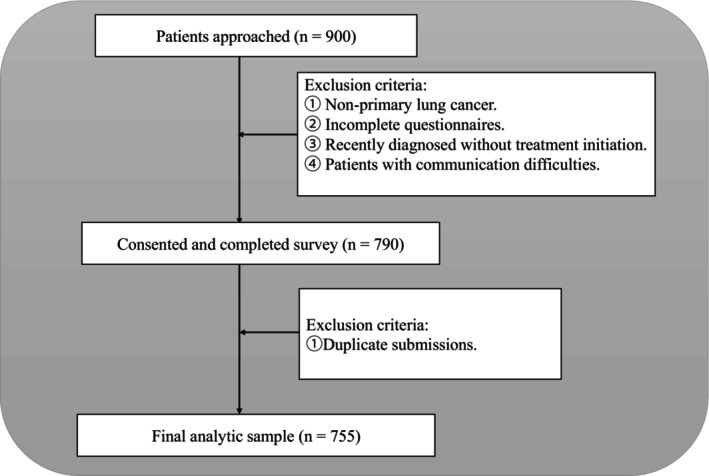
Flow diagram of participant recruitment and inclusion.

This cross‐sectional study utilized random sampling and was conducted at the Cancer Hospital of Shandong First Medical University from October to December 2023. The study adhered strictly to inclusion and exclusion criteria, including only hospitalized patients with lung cancer. Inclusion criteria were: (1) pathologically confirmed primary lung cancer; (2) currently receiving cancer treatment (including but not limited to chemotherapy, surgery, radiotherapy, targeted therapy, or immunotherapy); (3) provision of written informed consent. Exclusion criteria were: (1) patients who were not informed about their lung cancer diagnosis; (2) patients with hearing impairments or difficulties in verbal communication. Additionally, patients who submitted duplicate responses to the survey were excluded. A total of 755 cases were analyzed. A participant flow diagram illustrating the sampling and exclusion process is presented in Figure 1.

### 2.2 Study Procedure

The recruitment of participants for this study was methodically executed, beginning with the identification of potential subjects through the hospital's inpatient system. A team comprising clinical staff and research coordinators managed direct contact with eligible patients. The informed consent process was diligently overseen to guarantee that all participants thoroughly understood the study's purpose and methods before providing their written informed consent. Once consent was obtained, participants had the option to complete the survey either on paper during their hospital stay or via face‐to‐face interviews, accommodating those unable to complete the questionnaire independently.

A simple random sampling method was then used to select participants from this list. Each eligible patient was assigned a random number, and those with the lowest numbers were sequentially invited to participate until the required sample size was reached. The sampling and recruitment process was carried out by trained research staff independent of clinical care. This study adhered stringently to the ethical standards of the Declaration of Helsinki and received approval from the Ethics Committee of the Cancer Hospital of Shandong First Medical University (Approval No. SDTHEC‐2023009018). Throughout the study, stringent measures were enforced to safeguard the privacy and confidentiality of all participants. The design, data collection, and reporting processes conformed to the Strengthening the Reporting of Observational Studies in Epidemiology (STROBE) guidelines, ensuring transparency and rigor in the research.

### 2.3 Measures

#### 2.3.1 Socioeconomic Status (SES)

This study assessed socioeconomic status (SES) using a scoring method based on the classification by Torres et al. [21], which has also been widely adopted in Chinese public health and oncology research [22, 23]. The scoring system assigned values ranging from 1 to 5 for each indicator—household income, personal education level, and occupation—representing a spectrum from low to high. The total SES score for each participant was calculated as the sum of these values across the three indicators, with higher scores indicating a higher socioeconomic status. The criteria for scoring the measures of socioeconomic status are detailed in Table 1.

**TABLE 1:** Criteria for assigning measurement indicators of SES.

Family annual income (CNY)	Employment	Education	SEM score
< 10,000	Government/Institutional employee	No education	1
10,000 ~ 50,000	Enterprise worker/Industrial worker	Primary school	2
50,000 ~ 100,000	Self‐employed/Freelancer	Middle school	3
100,000 ~ 150,000	Farmer	High school/Technical school	4
> 150,000	Unemployed	College and above	5

#### 2.3.2 Comprehensive Score for Financial Toxicity (COST)

The Comprehensive Score for Financial Toxicity (COST), developed by de Souza et al. [24], assesses both direct and indirect economic impacts, including out‐of‐pocket expenses and income loss due to work incapacitation. Its adaptation for the Chinese context, carried out by Yu et al. [25], produced a version with proven robust reliability among Chinese cancer patients. This instrument includes 11 components: one financial, two resource‐related, and eight that evaluate emotional impacts. Scores are determined using a five‐point Likert scale, from 0 (not at all) to 4 (very much), allowing the total score to range from 0 to 44. Higher scores denote lower financial toxicity. In our study, the instrument achieved a Cronbach's alpha of 0.85.

#### 2.3.3 Social Support Rating Scale (SSRS)

In this research, the level of social support for participants was quantified using the Social Support Rating Scale (SSRS) created by Xiao et al. [26]. This instrument is divided into three categories: objective support, subjective support, and support utilization, featuring a total of 10 questions. Questions 1–5 and 8–10 utilize a 4‐point Likert scale, where 1 denotes ‘not at all’ and 4 signifies ‘very much’. For questions 6 and 7, a response of ‘No source’ yields a score of 0, whereas ‘Have a source’ accrues 1 point per source identified. The overall possible scores on the SSRS can range from 12 to 66, with higher scores indicating enhanced levels of social support. The scale demonstrated good reliability in this study, evidenced by a Cronbach's alpha of 0.79.

#### 2.3.4 General Self‐Efficacy Scale (GSES)

The General Self‐Efficacy Scale (GSES), created by Zhang and Schwarzer [27], was employed to evaluate self‐efficacy, which reflects individuals' perceptions of their effectiveness in managing difficult situations and overcoming obstacles. We utilized the Chinese adaptation of the GSES, validated by Wang et al. [28], which has proven to be reliable. The scale consists of 10 items, each rated on a 4‐point scale. The overall score is computed by averaging these item scores, yielding a range from 1 to 4, with higher scores indicating stronger self‐efficacy. In our study, the instrument exhibited a Cronbach's alpha of 0.95.

#### 2.3.5 Covariates

In addition to the primary variables, we identified several potential confounders that may influence the effect of socioeconomic status (SEM) on the financial toxicity experienced by lung cancer inpatients. These covariates include age (in years), sex (1 = male, 2 = female), marital status (1 = married, 2 = widowed/divorced/unmarried), and residence (1 = rural, 2 = urban).

### 2.4 Statistic Analysis

The data for this study were analyzed using R 4.3.1. Initially, a descriptive statistical analysis of the variables was conducted, describing metric data with mean (*M*) and standard deviation (SD), and count data with percentages or proportions. Subsequently, the correlation between the research variables was examined. Finally, a chained mediation analysis was performed using Model 6 in the R PROCESS 4.0 macro. After controlling for covariates, the level of socioeconomic status (SES) was designated as the independent variable (*X*), with financial toxicity as the outcome variable (*Y*). Social support (M1) and general self‐efficacy (M2) were considered as the mediating variables. The mediation effects were tested and confidence intervals were estimated using the Bootstrap method with 5000 resamples. The significance of the direct, indirect, and total effects was determined based on whether the confidence intervals included zero. Differences were considered statistically significant at *p* < 0.05.

## 3 Results

### 3.1 Descriptive Statistics and Correlation Analysis

The analysis included a total of 755 lung cancer patients, comprising 521 males (69.01%) and 234 females (30.99%), with a mean age of 62.76 (SD = 9.40). The average financial toxicity score was 17.81 (SD = 8.45), detailed in Table 2. In this cohort, the COST score positively correlated with scores for socio‐economic status, social support, and self‐efficacy. The correlations among study variables are presented in Table 3.

**TABLE 2:** Descriptive statistical analysis (*n* = 755).

Characteristic	*N* (%)	COST score	*p*	*M* ± SD	Range
		*M* ± SD			
Age (year)				62.76 (9.40)	28 ~ 92
Gender					
Male	521 (69.01%)	18.01 (8.63)	0.331		
Female	234 (30.99%)	17.38 (8.05)			
Marital					
Married	710 (94.04%)	17.73 (8.41)	0.342		
Widowed/divorced/unmarried	45 (5.96%)	19.07 (9.09)			
Residence					
Rural	499 (66.09%)	16.73 (7.81)	< 0.001		
Urban	256 (33.91%)	19.92 (9.24)			
Education					
No education	72 (9.54%)	17.36 (8.29)	< 0.001		
Primary school	226 (29.93%)	16.60 (8.38)			
Middle school	265 (35.10%)	17.75 (8.24)			
High school/Technical school	135 (17.88%)	18.48 (8.20)			
College and above	57 (7.55%)	21.86 (9.35)			
Employment					
Government/Institutional employee	91 (12.05%)	23.23 (9.91)	< 0.001		
Enterprise worker/Industrial worker	184 (24.37%)	18.32 (8.51)			
Self‐employed/Freelancer	49 (6.49%)	18.04 (8.97)			
Farmer	220 (29.14%)	16.40 (8.17)			
Unemployed	211 (27.95%)	16.45 (6.82)			
Family annual income (CNY)					
< 10,000	214 (28.34%)	16.20 (7.33)	< 0.001		
10,000 ~ 50,000	307 (40.66%)	15.49 (7.65)			
50,000 ~ 100,000	147 (19.47%)	20.01 (8.05)			
100,000 ~ 150,000	37 (4.90%)	23.14 (7.70)			
> 150,000	50 (6.62%)	28.58 (7.98)		7.68 (2.80)	3 ~ 15
SSRS score				39.11 (6.93)	15 ~ 58
GSES score				2.22 (0.77)	1 ~ 4
COST score				17.81 (8.45)	0 ~ 44

**TABLE 3:** Correlation analysis of variables.

Variable	1	2	3	4	5	6	7
1. Age	—						
2. Gender	−0.11**	—					
3. Marital	0.22***	0.04	—				
4. Residence	−0.02	−0.03	0.01	—			
5. SEM_score	−0.08*	−0.13***	−0.02	0.57***	—		
6. Support	−0.09*	−0.03	−0.16***	0.12**	0.26***	—	
7. Gsesscore	0.00	−0.07	0.02	0.06	0.22***	0.28***	—
8. Ftscore	0.14***	−0.03	0.04	0.18***	0.31***	0.19***	0.40***

* *p* < 0.05.

** *p* < 0.01.

*** *p* < 0.001.

### 3.2 Analysis of Chain Mediation Effects

Based on the results of descriptive and correlational analyses, this study employed a chain mediation model while controlling for age, gender, marital status, and residence to further investigate the mediating roles of social support and self‐efficacy between socioeconomic status and financial toxicity. As shown in Table 4, SES had a positive effect on financial toxicity among lung cancer patients (*β* = 0.679, *p* < 0.001), indicating that higher socioeconomic status is associated with higher total scores on the COST, reflecting lower levels of financial toxicity. Including the variables of social support and self‐efficacy, the results demonstrated that SES positively predicts social support (*β* = 0.686, *p* < 0.05) and self‐efficacy (*β* = 0.055, *p* < 0.001). Higher levels of social support were significantly correlated with higher levels of self‐efficacy (*β* = 0.028, *p* < 0.05). However, the association between social support and financial toxicity was not statistically significant (*β* = 0.057, *p* > 0.05). Self‐efficacy had a positive effect on financial toxicity (*β* = 3.706, *p* < 0.001), suggesting that higher self‐efficacy levels are associated with higher total scores on the COST, indicating lower financial toxicity.

**TABLE 4:** Regression analysis of socioeconomic status, social support, and self‐efficacy on financial toxicity.

Characteristic	COST score	SSRS score	GSES score	COST score
Intercept	17.678***	39.539***	1.170***	17.404***
Age	0.148***	−0.027	0.002	0.146***
Gender	0.460	0.067	−0.061	0.677
Marital	0.258	−4.148	0.201	0.184
Residence	−0.069	−0.585	−0.143*	0.554
SEM score	0.992***	0.686***	0.055***	0.679***
SSRS score			0.028***	0.057
GSES score				3.706***

Abbreviations: COST score, Comprehensive Score for Financial Toxicity; GSES score, General Self‐Efficacy Scale; SEM score, socioeconomic status score; SSRS score, Social Support Rating Scale score.

* *p* < 0.05.

*** *p* < 0.001.

**FIGURE 2:**
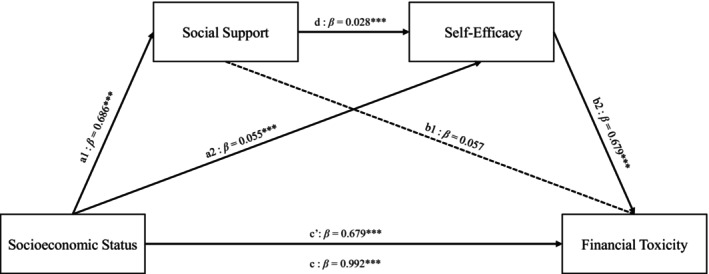
A model of the chain‐mediated effects of social support and self‐efficacy between socioeconomic status and financial toxicity.

Using Model 6 of the R PROCESS macro with covariates, this study examined the mediating roles of social support levels and general self‐efficacy in the relationship between socioeconomic status and financial toxicity. As shown in Table 5, the total effect was significant (effect = 0.992, *p* < 0.001), as were both the direct effect (effect = 0.679, *p* < 0.001) and the indirect effect (effect = 0.314, *p* < 0.001). The mediating effect of social support levels on the relationship between socioeconomic status and financial toxicity was not significant (effect = 0.203, *p* = 0.194), whereas general self‐efficacy demonstrated a significant mediating effect (effect = 0.203, *p* < 0.001). Additionally, the chain mediation effect of social support levels and general self‐efficacy on the relationship between socioeconomic status and financial toxicity was significant (effect = 0.072, *p* < 0.001). The chain mediation effect of social support levels and general self‐efficacy between SES and financial toxicity is illustrated in Figure 2.

**TABLE 5:** Analysis of chain mediating effects.

	Effect	SE	*p*	Low CI	Upper CI	Beta	Proportion (%)
Indirect effect	0.314	0.061	< 0.001	0.023	0.436	0.104	0.317
Ind_X_M1_Y	0.039	0.030	0.194	−0.018	0.102	0.013	0.039
Ind_X_M2_Y	0.203	0.051	< 0.001	0.110	0.307	0.067	0.205
Ind_X_M1_M2_Y	0.072	0.017	< 0.001	0.042	0.110	0.024	0.073
Direct	0.679	0.123	< 0.001	0.438	0.916	0.225	0.684
Total effect	0.992	0.129	< 0.001	0.739	1.242	0.329	

*Note:* Ind_X_M1_Y = Socioeconomic status → Social support → Financial toxicity; Ind_X_M2_Y = Socioeconomic status → Self‐efficacy → Financial toxicity; Ind_X_M1_M2_Y = Socioeconomic status → Social support → Self‐efficacy → Financial toxicity.

## 4 Discussion

Lung cancer represents a significant malignancy in China, not only leading in incidence but also as the leading cause of cancer‐related mortality [1, 29]. The high costs associated with lung cancer treatment, along with indirect costs such as lost productivity, impose severe financial toxicity on patients and their families, profoundly affecting their lives [30]. Thus, it is essential to consider these impacts when devising treatment and management strategies for lung cancer, actively seeking interventions to alleviate the economic burden on families. To our knowledge, few studies in mainland China have examined how socioeconomic status, social support, and self‐efficacy jointly relate to financial toxicity among hospitalized lung cancer patients. Our findings indicate a negative correlation between socioeconomic status and the severity of financial toxicity. Furthermore, social support and self‐efficacy are demonstrated to exhibit a chain mediation effect between socioeconomic status and financial toxicity.

Our research demonstrates a significant correlation between socioeconomic status and financial toxicity; specifically, higher socioeconomic status is associated with lower levels of financial toxicity experienced by patients. Patients with higher socioeconomic status, characterized by greater educational attainment, higher household income, and superior occupational status, tend to possess enhanced capacities to manage health risks. During cancer treatment, these patients typically face lighter economic burdens and less financial toxicity, corroborating existing literature that identifies income, education, and occupational status as protective factors against financial toxicity [31, 32]. Socioeconomic status serves as a comprehensive indicator that reflects both an individual's and their family's overall capacity to handle health challenges. This aspect is particularly crucial for cancer patients, as it may help buffer the negative impacts of financial toxicity, thereby enhancing the sustainability of treatment and improving overall quality of life [33]. The theory of health inequalities suggests that unequal social structures indirectly impact individual health outcomes through variables such as education, income, and occupational status. These factors often expose individuals with lower socioeconomic status to greater health risks and fewer medical resources, thereby increasing their vulnerability to financial toxicity [34]. Specifically, lung cancer patients with lower socioeconomic status may experience significant disparities in pre‐illness conditions, treatment durations, and survival rates compared to their counterparts with higher socioeconomic status. These patients often have poorer lifestyle habits, greater exposure to disease risk factors, and a reduced capacity to manage these risks, thereby exhibiting less resilience against the economic strains associated with cancer treatment.

Initially, self‐efficacy mediates independently between socioeconomic status and financial toxicity, underscoring the pivotal role of psychological factors in perceiving economic stress. Specifically, lower socioeconomic status is often associated with diminished self‐efficacy, which in turn correlates with higher financial toxicity, characterized by rising medical costs and heightened psychological stress [35]. These findings align with Bandura's theory of self‐efficacy, which posits self‐efficacy as a critical psychological resource for tackling challenges and overcoming adversities [36]. Therefore, alleviating medical financial strain should consider an integrated approach that includes both economic assistance and psychological support [37]. For populations with lower socioeconomic status, in addition to necessary financial aid, strategies to reduce financial toxicity should also enhance self‐efficacy through interventions such as self‐efficacy training and psychological counseling services. It is recommended that government and relevant agencies not only provide financial support but also bolster self‐efficacy through education and community services, thus mitigating financial toxicity on a broader societal level [35].

Secondly, our study did not identify a significant mediating effect of social support between socioeconomic status and financial toxicity, a finding inconsistent with some previous research. Furthermore, multivariate regression analysis revealed no significant correlation between social support and financial toxicity. Several factors may account for these results. In certain contexts, even when social support is prevalent, its practical efficacy in alleviating financial toxicity might be lower than expected [38]. This could be attributed to the inadequate capability of traditional forms of social support to address the fundamental economic challenges faced by hospitalized lung cancer patients under severe economic stress [39]. Additionally, the receptivity to social support can vary widely depending on cultural backgrounds, personal traits, or previous experiences. For instance, in cultural contexts such as China, individuals with strong values of pride, self‐reliance, or reluctance to “burden others” may be less inclined to seek or accept help, potentially diminishing the role of social support in mitigating financial toxicity [40]. Moreover, it is also possible that the measurement instrument used in this study—the Social Support Rating Scale (SSRS)—primarily captures general psychosocial support rather than financial or instrumental support, which may have limited its ability to detect associations with financial toxicity. Another possibility is that the relationship between social support and financial toxicity may be moderated by unmeasured individual or contextual factors, such as patients' health literacy, coping style, or prior access to formal financial counseling. Notably, although the path from social support to self‐efficacy was statistically significant, the effect size was very small (*β* = 0.028), raising concerns about its clinical significance. This suggests that while social support may contribute modestly to psychological adjustment, it may not play a central role in reducing financial toxicity in this context.

Finally, in this study, we found that when social support was considered as a standalone mediator, its statistical impact was not significant. However, when social support was integrated with self‐efficacy to construct a chained mediation model, both factors exhibited significant mediating effects between socioeconomic status and financial toxicity. This discovery underscores a complex psychosocial interaction mechanism: social support may indirectly alleviate financial toxicity by enhancing individual self‐efficacy [41]. Understanding this chained mechanism not only deepens our knowledge of the dynamics underlying financial toxicity but also suggests that future preventive measures should simultaneously strengthen social support and individual psychological capacities. Moreover, these findings provide significant expansions to the theoretical models of financial toxicity, recommending that future research should further explore the roles of social support and self‐efficacy across various manifestations of financial toxicity and investigate the efficacy of this model across different socioeconomic groups and cultural contexts.

## 5 Implications

This study found that socioeconomic status significantly influences the financial toxicity experienced by lung cancer patients, exhibiting a negative effect; higher socioeconomic status is associated with less financial toxicity. Further, we explored the chained mediating roles of social support and self‐efficacy. While social support alone did not reach statistical significance as a mediator, its combination with self‐efficacy showed a significant chained mediating effect between socioeconomic status and financial toxicity. This highlights the critical role of psychosocial factors in alleviating financial toxicity. Based on these findings, we propose the following interventions: Enhancing self‐efficacy is crucial for mitigating financial toxicity in lung cancer patients. Healthcare providers and community organizations should design and implement educational and psychological support programs aimed at boosting patients' self‐efficacy, such as self‐management education and goal‐setting training [42]. Strengthening social support, although social support alone did not show a significant effect, its combined action with self‐efficacy significantly reduces financial toxicity. Therefore, patients' social support networks should be strengthened, and their psychological adaptability enhanced through training and educational initiatives, such as community activities, support groups, or online communities [43]. Designing comprehensive intervention measures, government and healthcare policymakers should consider multidimensional intervention strategies that integrate financial aid with psychosocial support, including providing financial assistance, medical cost subsidies, and psychological and social support services, to fully meet patients' needs [44]. Future research directions, it is recommended that future studies continue to explore the roles of social support and self‐efficacy across different cancer types and populations, especially under varying socioeconomic and cultural backgrounds. This will help deepen our understanding of the mechanisms underlying financial toxicity, thereby enabling the design of more effective intervention measures.

## 6 Limitation

This study has several limitations. First, we employ a cross‐sectional design, which precludes establishing causal relationships between variables. Although we observed correlations between socioeconomic status and financial toxicity in lung cancer patients, these associations should not be interpreted as causal. The findings represent associative trends rather than direct effects. Future research should utilize longitudinal or experimental designs, with prospective data collection and analysis, to more thoroughly examine the mechanisms and temporal relationships underlying financial toxicity during cancer treatment. Additionally, our data primarily rely on patient self‐reporting, which may be subject to recall and social desirability biases that could affect the accuracy and reliability of the data. Although the participants were recruited from a single hospital, which may limit the representativeness of the sample, this institution is the largest tertiary cancer center in Shandong Province and receives a diverse patient population from across the region, partially mitigating concerns about regional bias. Therefore, future studies should adopt a multicenter design to enhance the generalizability and applicability of the findings. Furthermore, although a random sampling method was used, the possibility of selection bias cannot be entirely ruled out, particularly due to the requirement for informed consent and the potential exclusion of patients who were unwilling or unable to participate.

## 7 Conclusion

In summary, our study uncovers that hospitalized lung cancer patients are facing significant financial toxicity. Importantly, the results indicate that higher socioeconomic status was associated with financial toxicity in these patients. Furthermore, social support and general self‐efficacy were identified as chained mediating mechanisms linking socioeconomic status with financial toxicity. Specifically, individuals with higher socioeconomic status tend to receive more social support, which in turn is associated with stronger self‐efficacy during cancer treatment, and this combination is associated with lower levels of financial toxicity. Future initiatives may consider enhancing social support for patients with lower socioeconomic status, as a potential approach to strengthening self‐efficacy and alleviating the adverse impacts of financial toxicity.

## Author Contributions

**Yan Liu:** methodology, software, data curation, validation, formal analysis, writing – original draft, writing – review and editing, investigation, supervision. **Pengfei Li:** conceptualization, methodology, validation, supervision, project administration, resources. **Boyu Liu:** methodology, software, data curation, investigation, visualization, resources. **Yuantao Qi:** conceptualization, methodology, software, investigation, validation. **Weimin Guan:** investigation, data curation, validation, visualization. **Nan Zhang:** funding acquisition, project administration, resources. **Youhua Lu:** project administration, resources, conceptualization, funding acquisition.

## Ethics Statement

This study was approved by the Ethics Committee of the Cancer Hospital of Shandong First Medical University (SDTHEC‐2023009018) and all patients provided written informed consent for their participation in the study.

## Conflicts of Interest

The authors declare no conflicts of interest.

## Data Availability

The datasets generated during and/or analyzed during the current study are available from the corresponding author on reasonable request.

## References

[1] B. Han , R. Zheng , H. Zeng , et al., “Cancer Incidence and Mortality in China, 2022,” Journal of the National Cancer Center 4 (2024): 47–53, 10.1016/j.jncc.2024.01.006. 39036382 PMC11256708

[2] C. Liu , J. Shi , H. Wang , et al., “Population‐Level Economic Burden of Lung Cancer in China: Provisional Prevalence‐Based Estimations, 2017–2030,” Chinese Journal of Cancer Research 33 (2021): 79–92, 10.21147/j.issn.1000-9604.2021.01.09. 33707931 PMC7941692

[3] Y. Jia , W. Jiang , B. Yang , S. Tang , and Q. Long , “Cost Drivers and Financial Burden for Cancer‐Affected Families in China: A Systematic Review,” Current Oncology 30 (2023): 7654–7671, 10.3390/curroncol30080555. 37623036 PMC10453571

[4] M. Su , J. Lao , N. Zhang , et al., “Financial Hardship in Chinese Cancer Survivors,” Cancer 126 (2020): 3312–3321, 10.1002/cncr.32943. 32396242

[5] X. Wei , H. Sun , J. Zhuang , et al., “Cost‐Effectiveness Analysis of CYP2D6*10 Pharmacogenetic Testing to Guide the Adjuvant Endocrine Therapy for Postmenopausal Women With Estrogen Receptor Positive Early Breast Cancer in China,” Clinical Drug Investigation 40 (2020): 25–32, 10.1007/s40261-019-00842-0. 31559573

[6] S. Liu , M. Su , N. Yao , et al., “Employment Changes Among Chinese Family Caregivers of Long‐Term Cancer Survivors,” BMC Public Health 20 (2020): 1787, 10.1186/s12889-020-09922-9. 33238976 PMC7690119

[7] S. Y. Zafar , J. M. Peppercorn , D. Schrag , et al., “The Financial Toxicity of Cancer Treatment: A Pilot Study Assessing Out‐of‐Pocket Expenses and the Insured Cancer Patient's Experience,” Oncologist 18 (2013): 381–390, 10.1634/theoncologist.2012-0279. 23442307 PMC3639525

[8] S. Y. Zafar and A. P. Abernethy , “Financial Toxicity, Part I: A New Name for a Growing Problem,” Oncology 27 (2013): 80–81. 23530397 PMC4523887

[9] H. R. Abrams , S. Durbin , C. X. Huang , et al., “Financial Toxicity in Cancer Care: Origins, Impact, and Solutions,” Translational Behavioral Medicine 11 (2021): 2043–2054, 10.1093/tbm/ibab091. 34850932

[10] D. Ürek and Ö. Uğurluoğlu , “Predictors of Financial Toxicity and Its Associations With Health‐Related Quality of Life and Treatment Non‐Adherence in Turkish Cancer Patients,” Support Care Cancer 30 (2022): 865–874, 10.1007/s00520-021-06491-4. 34392415

[11] C. Barakat and T. Konstantinidis , “A Review of the Relationship Between Socioeconomic Status Change and Health,” International Journal of Environmental Research and Public Health 20 (2021): e6249, 10.3390/ijerph20136249. PMC10341459 37444097

[12] J. Yong and O. Yang , “Does Socioeconomic Status Affect Hospital Utilization and Health Outcomes of Chronic Disease Patients?,” European Journal of Health Economics 22 (2021): 329–339, 10.1007/s10198-020-01255-z. 33389255

[13] M. de la Cruz and M. O. Delgado‐Guay , “Financial Toxicity in People Living With Advanced Cancer: A New, Deadly, and Poorly Addressed Effect of Cancer and Necessary Treatment,” Seminars in Oncology Nursing 37 (2021): 151171, 10.1016/j.soncn.2021.151171. 34294500

[14] D. Ozdemir and F. Tas Arslan , “An Investigation of the Relationship Between Social Support and Coping With Stress in Women With Breast Cancer,” Psycho‐Oncology 27 (2018): 2214–2219, 10.1002/pon.4798. 29905003

[15] D. E. Kelley , E. E. Kent , K. Litzelman , M. A. Mollica , and J. H. Rowland , “Dyadic Associations Between Perceived Social Support and Cancer Patient and Caregiver Health: An Actor‐Partner Interdependence Modeling Approach,” Psychooncology 28 (2019): 1453–1460, 10.1002/pon.5096. 30993811 PMC6749989

[16] G. L. Smith , M. P. Banegas , C. Acquati , et al., “Navigating Financial Toxicity in Patients With Cancer: A Multidisciplinary Management Approach,” CA: A Cancer Journal for Clinicians 72 (2022): 437–453, 10.3322/caac.21730. 35584404 PMC12994614

[17] M. Peters , C. M. Potter , L. Kelly , and R. Fitzpatrick , “Self‐Efficacy and Health‐Related Quality of Life: A Cross‐Sectional Study of Primary Care Patients With Multi‐Morbidity,” Health and Quality of Life Outcomes 17 (2019): 37, 10.1186/s12955-019-1103-3. 30764833 PMC6376655

[18] G. Sadigh , N. Lava , J. Switchenko , et al., “Patient‐Reported Financial Toxicity in Multiple Sclerosis: Predictors and Association With Care Non‐Adherence,” Multiple Sclerosis Journal 27 (2021): 453–464, 10.1177/1352458520913977. 32808562

[19] X. Yuan , X. Zhang , J. He , and W. Xing , “Interventions for Financial Toxicity Among Cancer Survivors: A Scoping Review,” Critical Reviews in Oncology/Hematology 192 (2023): 104140, 10.1016/j.critrevonc.2023.104140. 37739147

[20] D.‐F. Wang , Y.‐N. Zhou , Y.‐H. Liu , et al., “Social Support and Depressive Symptoms: Exploring Stigma and Self‐Efficacy in a Moderated Mediation Model,” BMC Psychiatry 22 (2022): 117, 10.1186/s12888-022-03740-6. 35168584 PMC8845403

[21] V. A. Torres , J. M. Ashford , E. Wright , et al., “The Impact of Socioeconomic Status (SES) on Cognitive Outcomes Following Radiotherapy for Pediatric Brain Tumors: A Prospective, Longitudinal Trial,” Neuro‐Oncology 23 (2021): 1173–1182, 10.1093/neuonc/noab018. 33543269 PMC8248851

[22] R. MasoudiSani , A. Taheri , and N. Babakhani , “The Mediating Role of Guilt in the Relationship Between Suffering and Depression Among Caregivers of Elderly Patients With Life‐Threatening Diseases,” Journal of Assessment in Research, Applications, and Counseling 6 (2022): 129–134, 10.61838/kman.jarac.6.2.16.

[23] S. Zhang , H. Wang , B. Liu , J. Yu , and Y. Gao , “Socioeconomic Status Index Is an Independent Determinant of Breast Cancer Screening Practices: Evidence From Eastern China,” PLoS One 17 (2022): e0279107, 10.1371/journal.pone.0279107. 36516181 PMC9749974

[24] J. A. de Souza , B. J. Yap , F. J. Hlubocky , et al., “The Development of a Financial Toxicity Patient‐Reported Outcome in Cancer: The COST Measure,” Cancer 120 (2014): 3245–3253, 10.1002/cncr.28814. 24954526

[25] H.‐H. Yu , Z.‐F. Yu , H. Li , H. Zhao , J. M. Sun , and Y. Y. Liu , “The COmprehensive Score for Financial Toxicity in China: Validation and Responsiveness,” Journal of Pain and Symptom Management 61 (2021): 1297–1304.e1, 10.1016/j.jpainsymman.2020.12.021. 33412268

[26] S. Xiao , “Theoretical Basis and Research Application of the Social Support Assessment Scale,” Journal of Clinical Psychiatry 4 (1994): 98.

[27] A. Luszczynska , M. Tryburcy , and R. Schwarzer , “Improving Fruit and Vegetable Consumption: A Self‐Efficacy Intervention Compared With a Combined Self‐Efficacy and Planning Intervention,” Health Education Research 22 (2007): 630–638, 10.1093/her/cyl133. 17060349

[28] C. K. Wang , “Evidences for Reliability and Validity of the Chinese Version of General Self‐Efficacy Scale,” Journal of Applied Psychology 7 (2004): 37–40, 10.3969/j.issn.1006-6020.2001.01.007.

[29] F. Bray , M. Laversanne , H. Sung , et al., “Global Cancer Statistics 2022: GLOBOCAN Estimates of Incidence and Mortality Worldwide for 36 Cancers in 185 Countries,” CA: A Cancer Journal for Clinicians 74 (2024): 229–263, 10.3322/caac.21834. 38572751

[30] N. Deboever , M. Eisenberg , W. L. Hofstetter , et al., “Financial Toxicity in Patients With Resected Lung Cancer,” Annals of Surgery 278 (2023): 1038–1044, 10.1097/SLA.0000000000005926. 37249193

[31] F. Mols , B. Tomalin , A. Pearce , B. Kaambwa , and B. Koczwara , “Financial Toxicity and Employment Status in Cancer Survivors. A Systematic Literature Review,” Support Care Cancer 28 (2020): 5693–5708, 10.1007/s00520-020-05719-z. 32865673 PMC7686183

[32] A. Pearce , B. Tomalin , B. Kaambwa , et al., “Financial Toxicity Is More Than Costs of Care: The Relationship Between Employment and Financial Toxicity in Long‐Term Cancer Survivors,” Journal of Cancer Survivorship 13 (2019): 10–20, 10.1007/s11764-018-0723-7. 30357537

[33] K.‐X. Yu , W.‐J. Yuan , C.‐H. Huang , et al., “Socioeconomic Deprivation and Survival Outcomes in Patients With Colorectal Cancer,” American Journal of Cancer Research 12 (2022): 829–838. 35261805 PMC8899994

[34] K. L. Frohlich and T. Abel , “Environmental Justice and Health Practices: Understanding How Health Inequities Arise at the Local Level,” Sociology of Health & Illness 36 (2014): 199–212, 10.1111/1467-9566.12126. 24372359

[35] B. Thom and C. Benedict , “The Impact of Financial Toxicity on Psychological Well‐Being, Coping Self‐Efficacy, and Cost‐Coping Behaviors in Young Adults With Cancer,” Journal of Adolescent and Young Adult Oncology 8 (2019): 236–242, 10.1089/jayao.2018.0143. 30817217 PMC6588118

[36] M. M. Gebauer , N. McElvany , O. Köller , and C. Schöber , “Cross‐Cultural Differences in Academic Self‐Efficacy and Its Sources Across Socialization Contexts,” Social Psychology of Education 24 (2021): 1407–1432, 10.1007/s11218-021-09658-3.

[37] Z. Rezaie , V. Kohpeima Jahromi , V. Rahmanian , and N. Sharifi , “The Effect of Educational Intervention Based on the Self‐Efficacy Theory of High School Students in Adopting Preventive Behaviors of COVID‐19,” Journal of Education Health Promotion 11 (2022): 383, 10.4103/jehp.jehp_274_22. 36618451 PMC9818692

[38] A. Chan , Y. Ke , M. Tanay , et al., “Financial Toxicity in Cancer Supportive Care: An International Survey,” JCO Global Oncology 10 (2024): e2400043, 10.1200/GO.24.00043. 38959449

[39] S. Zang , H. Zhan , L. Zhou , and X. Wang , “Research on Current Curative Expenditure Among Lung Cancer Patients Based on the “System of Health Accounts 2011”: Insights Into Influencing Factors,” Journal of Cancer 10 (2019): 6491–6501, 10.7150/jca.34891. 31777579 PMC6856899

[40] V. Pourmand , K. A. Lawley , and B. J. Lehman , “Cultural Differences in Stress and Affection Following Social Support Receipt,” PLoS One 16 (2021): e0256859, 10.1371/journal.pone.0256859. 34499676 PMC8428676

[41] A. N. Ehsan , C. A. Wu , A. Minasian , et al., “Financial Toxicity Among Patients With Breast Cancer Worldwide,” JAMA Network Open 6 (2022): e2255388, 10.1001/jamanetworkopen.2022.55388. PMC9909501 36753274

[42] Z.‐L. Zhang , Z. Xu , S.‐K. Yang , J.‐G. Huang , F.‐M. Huang , and Y.‐M. Shi , “Influence of Financial Toxicity on the Quality of Life in Lung Cancer Patients Undergoing Immunotherapy: The Mediating Effect of Self‐Perceived Burden,” Cancer Management and Research 16 (2024): 1077–1090, 10.2147/CMAR.S470862. 39220814 PMC11365491

[43] M. F. Miller , J. S. Olson , K. Doughtie , A. K. Zaleta , and K. P. Rogers , “The Interplay of Financial Toxicity, Health Care Team Communication, and Psychosocial Well‐Being Among Rural Cancer Patients and Survivors,” Journal of Rural Health 40 (2024): 128–137, 10.1111/jrh.12779. 37449966

[44] C. Brix , C. Schleussner , J. Füller , B. Roehrig , T. G. Wendt , and B. Strauss , “The Need for Psychosocial Support and Its Determinants in a Sample of Patients Undergoing Radiooncological Treatment of Cancer,” Journal of Psychosomatic Research 65 (2008): 541–548, 10.1016/j.jpsychores.2008.05.010. 19027442

